# Molecularly profiled trials: toward a framework of actions for the “nil actionables”

**DOI:** 10.1038/s41416-021-01423-9

**Published:** 2021-05-26

**Authors:** Allan Michael Jordan

**Affiliations:** Oncology Drug Discovery, Sygnature Discovery, BioCity, Nottingham, UK

**Keywords:** Drug development, Target identification, Drug development

## Abstract

The sequencing of tumour or blood samples is increasingly used to stratify patients into clinical trials of molecularly targeted agents, and this approach has frequently demonstrated clinical benefit for those who are deemed eligible. But what of those who have no clear and evident molecular driver? What of those deemed to have “nil actionable” mutations? How might we deliver better therapeutic opportunities for those left behind in the clamour toward stratified therapeutics? And what significant learnings lie hidden in the data we amass but do not interrogate and understand? This Perspective article suggests a holistic approach to the future treatment of such patients, and sets a framework through which significant additional patient benefit might be achieved. In order to deliver upon this framework, it encourages and invites the clinical community to engage more enthusiastically and share learnings with colleagues in the early drug discovery community, in order to deliver a step change in patient care.

## Background

In 2017, a 57-year-old female presented to the Christie Hospital in Manchester, UK, with lung adenocarcinoma, her disease having progressed through first-line chemotherapy. Enrolling on the TARGET trial,^1^ which aimed to compare the utility of circulating tumour DNA (ctDNA) and archival biopsy material for stratification with therapy, blood samples were taken and analysed by whole-exome sequencing. Her ctDNA profiling revealed an NRAS mutation, which was retrospectively confirmed in her archival tumour. Based on these findings, the molecular tumour board recommended enrolment on a Phase 1 trial of a first-in-human MEK inhibitor. On therapy, the patient demonstrated partial response with a 60% reduction in marker lesions and her disease remained controlled for 12 months.

As a member of the molecular tumour board for some of the early phases of the TARGET trial, successful alignment of patients with novel therapeutics naturally resulted in deep satisfaction, in that we had potentially delivered patient benefit and a meaningful life extension for patients who had failed to respond to standard of care therapies and whose future appeared bleak. In some cases, the ctDNA analysis established potential therapeutic options not evident from the sequencing of the archival biopsy, perhaps due to disease evolution or through inadequate sampling of the tumour’s intrinsic heterogeneity. When these unanticipated therapeutic options or measurable patient responses came, as they did for several patients, the satisfaction was even more profound.

As a translational scientist and drug discoverer, exposure to (and direct participation in) the decision making in these trials was both a career highlight, and a transformative experience. It is rare for practising bench chemists and biologists to be involved at the front line of patient care and experimental clinical medicine, and to see our novel drugs deliver tangible benefits in these patients was both motivating and humbling.

## A harsh reality

However, the over-arching lesson from these experiences was that, despite our efforts, such clinical successes are rare. As we toil away in the lab to deliver the next generation of experimental medicines into the clinic, we assume that our patient populations will be readily defined, easy to access, and both enrolment and response rates will be high. More so, perhaps, for targeted therapeutics, where clear genomic markers will signpost patients toward our trials, much as described in the case above.

It was an unwelcome surprise to discover that the harsh reality of the clinic was rather different. While stratification of patients in this setting is clearly of benefit to those with a defined actionable mutation and matched therapeutic option, the actual alignment rate remains disappointingly low. For our TARGET trial, in the first cohort of 100 patients, 41 were found to have a potentially actionable mutation, but only 11 received a matched therapy. Of these 11, only four achieved partial response. On paper, a 4% success rate. Cause for celebration for those who responded, clearly. But what of the 96 patients who received no obvious benefit from participation?

Two other molecularly profiled studies co-reported alongside TARGET. First, the WINTHER trial sought to incorporate RNA sequencing alongside genome profiling.^2^ Of 303 patients enrolled, around a third were matched to therapy. The addition of transcriptomics helped increase matching to therapy but only 8% of the consented cohort gained therapeutic benefit. Second, the i-Predict study sought molecularly matched combination therapies based on genetic profiling for 149 patients.^3^ Despite half the cohort being assigned a therapy, only 17 patients (11%) of the total cohort achieved any meaningful benefit.

More recently reported trials follow a similar worrisome pattern. The hugely ambitious Lung MATRIX trial, where an actionable mutation was a pre-requisite for trial entry, enrolled 5500 patients for screening, with over 2000 eligible for one of the 19 cohorts.^4^ Yet only 5% were entered onto a trial arm, and less than 1% remain on therapy. While several factors accounted for some of this attrition, such as continuation of standard-of-care therapies or patient deaths before trial initiation, it remains the case that we fail to match actionable mutations to targeted therapy for the majority of our cancer patients.

Taken together, these and other data suggest that only around 10% of patients with advanced disease have an identifiable and actionable mutation and will benefit from genetically informed or directed therapy.^5^ This bleak summary is no criticism of these trials, and such approaches clearly offer significant benefit to those patients approaching the end of their treatment options on standard of care therapy.

Instead, this summary suggests that there remains a significant, and often unasked, question emanating from these studies—what of the 90% of advanced cancer patients who do not have an obvious molecular driver and aligned treatment option? What of those patients with a “nil actionable” finding as a result of these profiling efforts? Clearly, some undefined mechanism is driving the oncological process in these patients. And while the positive outcomes for a modest number of patients in these trials deserve to be celebrated and should not be overlooked, these “nil actionable” patients often fade into the background as we assess the successes of our stratified trials. While many of these patients return to standard of care therapies or palliative care, from the standpoint of our stratified trials they remain a forgotten majority, lost in the percentages of those who fell outside successful alignment.

So, how do we, as a collective community, convert these “nil actionables”, into the “New Actionables*”*? How do we deliver novel therapeutics and novel stratification to offer improved care to those for whom other options are exhausted?

## A new path

For some, progress is already afoot. Novel RAS inhibitors entering the clinic offer opportunities to open new molecularly targeted cohorts in some previously inaccessible cancers. The introduction of assessments of microsatellite instability highlights patients who may respond positively to checkpoint inhibition. But the forgotten majority remain. Untreated, and untreatable with our stratified agents.

Back in the drug discovery labs, both small and large molecule delivery efforts continue to deliver more of the same—similar therapeutics against a limited portfolio of common targets and known actionable mutations. For example, a recent summary suggested there are approaching 2500 on-going clinical trials of anti-PD-1 therapies,^6^ either monotherapy or nuanced combinations with agents targeting over 240 other proteins. Given the sheer volume of different investigations, it is unsurprising that many are failing to adequately recruit due to saturation of the trial population. Meanwhile, targeted therapeutic research efforts remain largely focused upon the top twenty or so “favourite” kinases and pathways, despite the ready availability of compelling small-molecule therapeutics in trials or approved for these targets.^7^

Surely a new approach is needed. Perhaps the greatest learning from these trials is that, for many patients, single agent or combination targeted therapies will not stem the tide of advanced disease. And that our current genome profiling efforts alone are insufficient to match patient to therapy. Clearly, novel therapeutics and diagnostics, beyond the paradigms of current therapeutic modalities, are urgently required.

Now more than ever that approach can be driven from the clinic, and not the lab. Delivering patient benefit by learning directly from the hidden messages buried in the detailed genetic and epigenetic signatures of our patients, and not from the esoteric biological models in systems far removed from real-world patient biology, such as the uniform and monoclonal populations of cells exclusively driven by a single genetic driver mutation, or those with unnaturally high expression of oncogenic proteins, that we rely on to drive our drug discovery programmes.

Here, perhaps, lies our opportunity. Many times in our Tumour Board, our genetic analysis of non-actionable case reports would offer a tantalising selection of mutations which might just pre-dispose our patients to therapy, if only we knew which ones were important, and which were noise. But the devil remained in the detail, lost and unactioned. Inexplicable and obfuscated. Potential signatures of underlying tumour drive, and potential new drug targets, if only we were able to interrogate, identify, and explore them. Clearly, some of these mutations, and potentially other undetected molecular changes, were leading to the oncogenic drive that resulted in the patient’s malignancy, but the depth of our biological understanding, and our ability to decipher the activating pathways, was absent.

And so the nature of these drivers remained obscured, with no strategy to unravel the mutational landscape or the potential applicability of the plethora of aberrations we witnessed. And, obscured from understanding, the potential of these novel drivers to yield the next generation of drug targets for therapeutic exploitation, remained unexploited. To catalogue, categorise and understand the complex diversity and significance of these mutations was too daunting an endeavour for a small team to undertake, with any realistic hope of developing a thorough understanding.

## Toward a brave new world

Fast forward just a few years. One of the emerging themes from the COVID-19 pandemic has been the power of inter-disciplinary research and collaboration to accelerate both fundamental disease understanding and the progression of novel therapeutic concepts from biological hypothesis to clinical trial. There is no reason to suspect that, with the right will, this collaborative research framework could not be applied to the discovery of novel therapies for our patients.

Alongside our efforts to deploy molecular profiling to identify treatments for a limited number of patients, perhaps a closer and more interactive collaboration between the clinical community and bench scientists, bioinformaticians and drug discovery experts might allow a detailed exploration of the myriad of molecular signatures of unknown significance we see each week in our data reviews. While many of these findings will be coincidental, some will reveal new therapeutic vulnerabilities, stratifying with tumour type, or perhaps offering novel histology-agnostic tumour drivers for which new therapies may be delivered.

Such an exploration would require significantly deeper sequencing efforts, spanning not just the commonplace exome sequencing of known oncogenic drivers, but also encompassing other methodologies such as RNASeq to detect, for example, previously unknown gene fusions, alongside assessments of epigenetic states at both the protein and genomic level.

These deeply informative and holistic approaches are becoming increasingly prevalent across clinical centres and may yet reveal potential therapeutic options. But this treasure trove of information remains largely inaccessible, disparate, and unexploited. It seems inexplicable to me that we accrue this knowledge, then simply overlook or discard the overwhelming majority of it. Resigning ourselves to the *fait accompli* that our relatively limited arsenal of precision therapeutics offers no real option for many hundreds, if not thousands, of desperate patients. Rather than drawing upon this repository of proximal, patient-centric knowledge, we return to our simpler, more conventional and reductionist, molecular biology-derived approaches—ultimately to yield (for the most part) small-molecule therapeutics which fail to translate in the clinical setting. We deliver modest responses, generally in the order of just a few months,^8^ rather than durable, meaningful patient outcomes.

I believe we can, and must, do better.

The case for change, for a new approach to therapeutics discovery driven by detailed clinical context, is compelling. However, for many of us involved in the discovery of relevant targets and the advance of new therapeutics, this vital insight is difficult to come by. Access to clinical datasets, and detailed discussions around the true limitations of our current therapeutics, are obscured by a disconnect between the research and clinical communities that often seems impenetrable. But, as the latest pandemic has highlighted, with the right mindset this disconnect can be overcome to deliver real changes in knowledge and patient care.

## An ambitious framework for action

Here, then, is our challenge. How can we, and do we, come together more effectively, in order to pool our collective expertise and best engage with the immense collective learnings of the clinical community? How do we reveal the best, most imaginative, and most impactful ways of delivering new treatments, and new hopes, to our patient populations? Surely, the long-term vision for our molecularly profiled trials needs to change and broaden, not only to deliver benefit to those with current actionable mutations, but also to determinedly resolve to seek new and better ways to help those patients for whom “nil actionable” concludes their stratified therapeutic pathway?

A collective undertaking of this proposed magnitude will be long, arduous and expensive. To deeply interrogate our clinical datasets, to reveal these new opportunities, will be a complex and challenging endeavour. But I believe such an endeavour is feasible and is likely to reveal novel opportunities to deliver significant patient benefit.

Such an endeavour would be multi-faceted, with many hurdles and it would require a step change in present thinking. However, such an approach would seem to rest upon a small number of achievable key principles.

### *Deeper knowledge comes from deeper understanding*

At present, our stratification efforts tend to rely on a relatively limited assessment of a core panel of known genetic mutations. While whole-exome sequencing is now routine, much of the acquired data are not deeply interpreted or analysed. For many patients, for example, gene fusions are not routinely assessed due to their anticipated scarcity,^9^ potentially precluding access to known and effective therapies such as the selective TRK, ROS1 or RET inhibitors.^10–12^ Recent studies in breast cancer and other disease settings further highlight that detailed disease understanding demands approaches beyond just somatic DNA mutational assessment, and patient outcomes are improved if gene fusion analysis, RNA sequencing, protein and DNA epigenetic status are included.^13, 14^ Moreover, a knowledge of non-oncogene intrinsic weaknesses such as synthetic lethal gene pairings (see below), metabolic vulnerabilities and immune phenotyping are beginning to add further layers of detail, understanding and clarity.^15^ But the understanding, and the more effective interrogation of these tumours, demands a deeper, more uniform and holistic approach to the acquisition of clinical profiling data beyond “just” NGS datasets, correlating and cross-referencing new gene fusions and mutational families with epigenetic states, clinical outcomes and other patient-derived biological data. Disease-specific resources such as the S-CORT database,^16^ for colorectal cancer, assimilate clinical, transcriptomic, genetic, methylation and histological data for thousands of patients and demonstrate the feasibility of such an approach, demonstrating that curated, detailed information can indeed be made publicly accessible, and this treasure trove of information is already yielding novel stratification biomarkers and potential therapeutic opportunities.^17^

But these temporal studies only tell half the story. While they inform of the genetic status of our patients at time of treatment, or relapse, they miss the critical details of evolution in response to prior therapies or other selective pressures. They fail to inform whether a mutation has been passively carried forward, or actively selected for. And they ignore, at the genomic level, the history and experiences of the patient. In some cases, archival resections and biopsies may fill in some of this “back story”. But perhaps to move forward, we need to look backward at this evolution and be more proactive about the comprehensive longitudinal collection, cataloguing, sequencing and interrogation of the patient’s disease from the point of diagnosis. Clearly, such an approach would offer significant inconvenience, and potential clinical risk, for the patient. But perhaps the increased sensitivity and applicability of non-invasive genetic sampling, from blood or other more easily accessed biological material, offers the potential to gain a deeper understanding of this evolution, and the potential weaknesses it may instil.

### *Population-scale genetics will reveal novel actionable mutational families*

At present, many observed mutations in key oncogenes are classified as “nil actionable”, as our understanding is insufficient to determine their nature as passenger or driver mutations and align them with a known therapeutic agent. These somatic mutations arise as varied families of alterations, often clustered outside of known functional regions and their role in oncogenesis and tumour drive is rarely defined. However, I believe that wider and deeper bioinformatics analysis of the mutational landscape, supported by functional biology investigations can and will reveal mutational hotspot regions which will lead to actionable targets. A robust mechanism for collating and sharing population-level data on a global scale would allow identification of such potential hotspots, suggest areas for “wet” experimental investigation and, ultimately, novel points of therapeutic intervention. Indeed, such approaches are already yielding tantalising drug discovery activities,^18^ and suggesting both novel families of drug targets and innovative ways of targeting known cancer drivers such as EGFR and HER-2 which fall outside current stratification approaches and therapies.^19, 20^ These findings offer potential new options for significant patient populations and hint at the significant but unexploited promise of this framework.

### *Validation, validation, validation*

Swathes of genetic-level data can only be truly valuable if the dataset itself can be reduced to a meaningful and testable therapeutic hypothesis. For example, the powerful capabilities of gene editing techniques delivered the ability to investigate a myriad of genetic alterations to reveal novel drug targets, but it was the specific combination of these tools with a systematic and methodical experimental tour de force that resulted in public resources such as Project DRIVE, a detailed catalogue of context-specific cancer synthetic lethal dependencies.^21^ Identified dependencies were then validated independently in cells and in patient-derived materials, ultimately revealing hitherto unknown points of therapeutic intervention such as Werner Helicase and PTPN2,^22, 23^ with experimental agents now moving toward clinical investigation. Such platforms highlight the potential power of the holistic interplay of clinical data, basic biological experimentation, and translational drug discovery activities for the elucidation of novel stratification opportunities.

### *Data quality, and consistency is key*

Such a data compendium in and of itself is insufficient for the delivery of patient benefit, if it is not suitably curated and accessible. The history of data science suggests that far too often, such collections are acquired piecemeal in such a way that aggregation, annotation, and direct comparison are impossible across different sources. With sequencing information to date acquired using differing protocols and scope, and with data ownership often unclear or complicated by various payers (with molecularly profiled trials funded, for example, from governmental initiatives, research grant or industry-funded trials), collating and curating these data would not be a trivial exercise. Yet such an endeavour demands absolute integrity of our DNA and RNA sequence alignments, mutational and fusion calling algorithms, methylation array analysis etc. Fundamentally, consistency, quality and absolute precision are pivotal for the success of these analyses, particularly when considering the low-incidence somatic mutations which could be critical for the discovery of new cancer weaknesses. Further, the value of a well curated and accurate global data repository is significantly weakened without widespread, unfettered, and facile access, and the tools to allow ready and straightforward bioinformatic analysis and hypothesis generation. Databanks such as the Cancer Cell Line Encyclopaedia^24^ carry value, partly because they are well curated, but primarily because they can be accessed and queried without specialist knowledge or licenses, and because they sit behind well-crafted and data-driven interrogation platforms such as the DepMap Portal.^25^ It is this accessibility and open sharing of data which promotes hypothesis generation and experimentation, leading to novel therapeutics and patient benefit. Efforts toward this goal are already underway, with the sequencing data from 19 leading cancer centres now being captured within the AACR “Project GENIE” repository.^26^ This collaborative academic-industrial joint repository collates NGS data for up to 1000 genes, alongside limited therapeutic response and clinical outcome data for tens of thousands of patients in a curated and holistic manner,^27^ and interrogation of these data are beginning to yield new, treatable patient populations.^28^ As such, Project GENIE suggests an advanced blueprint for the underlying data and logistical framework needed to capture, collate and share patient-derived genetic and epigenetic data on a scale not limited to a one thousand gene NGS panel.

### *To work at all, we must all work together*

Our successes to date in the era of molecular profiling and patient stratification have frequently arisen through the direct and proactive interaction between basic biology, translational research and clinical expertise. The evolving story of treatments for lung cancer, for example, driven by EFGR mutations^29^ and ALK fusions,^30^ has been successful thanks to the interplay of basic pathway elucidation, drug discovery, imaginative clinical trials and the rapid feedback and learning around the almost inevitable appearance of clinical mutations and resistance,^31^ allowing second and third generation therapeutics to become rapidly available to patients and resulting in significantly improved outcomes. However, this broad, holistic interplay is disappointingly uncommon. As one peer reviewer of this manuscript insightfully noted, “many researchers will… focus on a specific signalling pathway or pathological process and will often not be interested at all with the broader view comprehensive profiling may reveal, and are thus interested in one mutation and not so much on how several mutations may collaborate to give rise to a clinically relevant phenotype”. This mindset often also typifies our translational activities, where new therapeutics are designed with narrow scope, to block the action of a single, specific driver mutation rather than potential families of related but unclassified mutations with a similar phenotype. Yes, our sequencing efforts need to broaden to encompass the wider biological context and disease relevance. But our basic disease understanding, and translational outlook as a whole, also needs to broaden to encompass and exploit the discoveries and biological weaknesses these activities will uncover, and to incorporate some of the significant genetic context, heterogeneity and complexity that is evident in real patients, but not in our simplified, routine pre-clinical caner models.^32^ For such an endeavour to be successful, research activities will need to focus upon rapid and robust target (de)validation, delivery of patient-relevant chemical tools, and the rapid clinical evaluation of such agents through adaptive and innovative basket-style trial approaches, likely in conjunction with key patient advocacy groups to help identify and recruit potentially rare patient subsets. Perhaps controversially, rapid and robust yes/no decision making will be much more critical than the more commonplace focussed and detailed pathway deconvolutions and investigations which have predominated in our thinking and experimentation over the past few decades.

### *Amicable and equitable access to therapeutics*

However detailed and thorough our understanding of the genetic weaknesses of a tumour, the presence and availability of a targeted therapeutic is clearly critical to patient benefit. But even when such a therapy exists, the ability to match drug to patient depends on a number of factors, including the licensing of the drug in that locality, or the availability of a local clinical trial. Whilst databases such as ClinicalTrials.gov^33^ and the ECMC trials database^34^ help in this respect, highlighting where and when such therapies might be available, drug access is often not guaranteed or convenient, due to location or trial access criteria. A more amicable and inclusive approach to drug access, allied with better matching of patient to experimental agent, would surely help convert further actionable mutations into real patient successes. And, whilst a “nil actionable” mutation is frustrating and saddening, the identification of an actionable mutation, while being unable to align this with a pre-existing therapeutic option, is surely an even greater tragedy and a failure of our systems?

### *Ambition, and funding*

Clearly, such an undertaking is ambitious and inter-disciplinary at a level beyond anything the cancer research community has undertaken before. And will be ferociously expensive to deliver. The data acquisition, the myriad of detailed sequencing activities for each enrolled patient alone, would be an order of magnitude beyond current activities. However, the global economic burden of cancer is estimated at around $1.16tn annually.^35^ On a financial basis alone, a compelling case for public-private partnership to both reduce this fiscal burden and to deliver a significant increase in both patient longevity and quality of life is not difficult to construct.

There will also be a challenge of ambition. “Wars on cancer” have come and gone,^36–38^ often with mixed appraisals of their success,^39^ and it is easy to dismiss undertakings of this magnitude as just “too complex” or “too challenging”. But activities such as the Cancer Research UK “Grand Challenges” demonstrate that, with the appropriate framework and mentality, ambitious challenges can be aggressively tackled,^40^ and appropriately funded. The success of recent public-private and academic-industrial partnerships such as DepMap and Project GENIE demonstrate that collaborative research and funding on a significant scale is feasible, translating emerging biological hypothesis into novel therapeutic delivery. And ambitious clinical trials such as MATRIX demonstrate that multiple stakeholders, across academic, patient, clinician and industrial sectors, can successfully work together to deliver ground-breaking approaches to cancer healthcare.^4^ While the current research and funding landscape is challenging against the backdrop of a global pandemic, the eagerness, willingness and determination to come together as a scientific community to deliver complex and challenging scientific endeavours for the benefit of patients has been an unexpected and powerful positive force for change in these unusual times. Such pre-competitive and collaborative enterprises may have seemed unthinkable just a few years ago, but they suggest a glimpse of what may be possible if we can capture and expand upon this new way of interactive collaboration. Such activities also suggest a framework for the economic impact assessment and funding mechanisms to deliver truly global impacts upon public health and the lives of cancer patients. (For comparison, the “once in a century” COVID-19 pandemic has been estimated to have a total global cost of $8-16tn,^41^ or the equivalent of less than a decade of cancer impact.)

## No patient left behind

Realising this objective will demand a more open and interactive dialogue across all our respective disciplines and areas of expertise. I believe we are all keen to listen, but also know that, from here in the lab, we are often not sure how to engage in that conversation. To break outside our silos and narrow foci and to engage in the wider debate that might just allow a step change in cancer care.

For that, I believe we must more actively engage with the collective guidance of the clinical community. To help us understand more deeply the clinical perspective, both at a detailed molecular level and that of the individual patient themselves. To understand where our lovingly crafted therapeutics are failing in the clinical setting. To collectively curate and interrogate the vast array of knowledge gained through the many, many molecularly targeted clinical trials to bring some much-needed light in the darkness of unmet clinical need. To deliver together the next generations of cancer therapeutics, with durable, well tolerated, and meaningful responses for patients in the latter stages of their cancer journey.

There would undoubtedly be many failures and blind alleys along the way. But I believe the time is right for us all to work together more openly and proactively. To change our mindset and to reveal new areas of cancer translational science. To more fully learn from the knowledge we routinely accrue, then overlook and dismiss. To turn the “non-actionable” outcomes into new, meaningful “actionables”. And to truly realise the vision of “no patient left behind”.

## Data Availability

Not applicable.
